# Anomalies in uncinate fasciculus development and social defects in preschoolers with autism spectrum disorder

**DOI:** 10.1186/s12888-019-2391-1

**Published:** 2019-12-16

**Authors:** Yun Li, Zhengbing Zhou, Chen Chang, Lu Qian, Chunyan Li, Ting Xiao, Xiang Xiao, Kangkang Chu, Hui Fang, Xiaoyan Ke

**Affiliations:** Child Mental Health Research Center, Nanjing Brain Hospital affiliated to Nanjing Medical University, No.264 Guangzhou Road, Nanjing, 210029 Jiangsu China

**Keywords:** Autism spectrum disorder, Diffusion tensor imaging, Tractography, Uncinate fasciculus, Longitudinal study

## Abstract

**Background:**

Individuals with autism spectrum disorder (ASD) have social interaction deficits and difficulties in emotional regulation. The neural substrates for these socio-affective deficits are not yet clear, but one potential candidate is maldevelopment of the uncinate fasciculus (UF), a white matter tract thought to be involved in socio-affective processing. However, the developmental trajectory of the UF in young children with social interaction deficits has not been examined. The present study was designed to describe the developmental growth trajectory of the UF and the relationships between UF development and social deficits in ASD.

**Methods:**

Eigenvalues of the UF were measured by diffusion tensor imaging (DTI)-based tractography in 37 children with ASD and 27 matched 2–3-year-old subjects with developmental delay (DD) at baseline (time 1) and at 2-year follow-up (time 2). Growth rates of the UF were compared between groups and associations with social deficit scores according to the Autism Diagnostic Interview-Revised (ADI-R) analyzed by Pearson’s correlations.

**Results:**

At time 1, axial diffusivity (AD) of the left UF was significantly larger in the ASD group than the DD group. At time 2, left UF fractional anisotropy (FA) was significantly higher and radial diffusivity (RD) significantly lower in the ASD group than the DD group. The rate of UF growth during this 2-year interval was faster in children with ASD than DD. Significant negative correlations were found between the rise in ADI-R social deficit measures and both right UF RD and left UF mean diffusivity (MD).

**Conclusions:**

Young children with ASD demonstrate UF overgrowth during the 2-year development period between 2 and 3 and 4–5 years of age, and this white matter abnormality is directly associated with the progression of social deficits.

**Trial registration:**

World Health Organization class I registered international clinical trial platform, ChiCTR-ROC-17012877.

## Background

Autism spectrum disorder (ASD) is a complex neurodevelopmental condition characterized by core impairments in social interaction and social communication as well as behavioral anomalies including repetitive, restrictive, and stereotyped patterns of interests and activities (American Psychiatric Association, 2013) [1]. To date, a growing number of studies have proven that there are often pronounced difficulties in regulating emotion across ASD patients [2–6].

One particularly plausible neural mechanism for the socio-affective deficits of ASD is abnormal development of the uncinate fasciculus (UF). The UF is known to be an essential connector, which bridge polar temporal regions to frontal lobe [7, 8]. Therefore, it is also regarded as a link between the basic emotion processing region and the area involving higher order processing [9]. Moreover, UF abnormities are associated with impaired socio-emotional processing and symptom severity in individuals with ASD [10].

The human brain follows a precise spatiotemporal pattern of maturation that begins with phylogenetically older posterior and inferior regions and then progressively extends to more anterior and superior regions [11, 12]. Magnetic resonance imaging (MRI) [13–18] and diffusion tensor imaging (DTI) studies [19–23] have suggested that concurrent changes in gray and white matter follow distinct developmental trajectories during brain maturation. Magnetic resonance imaging studies [24, 25] of brain anatomy in children with ASD have shown that developmental trajectory is atypical and includes early overgrowth, followed by growth retardation, and at an older age, potentially reduced brain volume. In addition, changes in the developmental trajectory of the ASD brain appear to differ among regions, with greater abnormalities in frontal and temporal lobes than parietal and occipital lobes.

Neuroimaging studies have also provided in vivo evidence of atypical neural network activity, connectivity, and white matter impairments in ASD [26]. White matter tracts form the structural foundation for brain connectivity by linking discrete gray matter regions into integrated neural circuits. Further, white matter tract connectivity regulates the speed and timing of activation across neural networks for optimal information processing [27–29]. Recent developmental DTI studies of healthy populations have reported that fractional anisotropy (FA) values of the UF, which are thought to reflect myelination, white matter organization, and fiber tract density, increase with age [30], whereas measures of local water diffusion such as the mean diffusivity (MD), axial diffusivity (AD), and radial diffusivity (RD) decrease [31].

Previous studies have uncovered a variety of white matter structures are altered in ASD [8, 32–34], which hinted the left UF may play a significant role in processing of socio-affective information [35]. The phenomenon of brain asymmetry during the process of brain maturation is critical to the normal development of emotion regulation as well as the functions of cognitive, sensory, and motor. In ASD, left UF could be exclusively affected by atypical brain maturation, and recent evidence has also shown that the left UF is specifically involved in socio-affective skills, including emotion regulation [36, 37].

However, there is conflicting evidence on UF microstructural atypicalities in individuals with autism [24]. Although several studies have reported decreased FA in the left [38] or bilateral UF [10], other studies have reported increases in FA [39] or no differences in FA compared to controls [40, 41]. It is possible that these different patterns are associated with distinct symptoms or behavioral characteristics of ASD. Alternatively, these differences for normal neural development may be age-dependent.

In reviewing previous studies, most UFs of ASD patients were found to have abnormal FA values; however, most of these studies were cross-sectional and so could not establish associations with age or symptom severity. A longitudinal study by Wolff and colleagues (2012) on infant siblings of autism patients, who are considered at higher risk of autism, reported that FA values in the UF were higher than in matched controls at 3 months of age but demonstrated a significantly smaller rate of change, resulting in significantly lower FA values in the UF and other white matter association tracts by 24 months [42]. Autism spectrum disorder is currently diagnosed at the age of two; thus, a two-year longitudinal follow-up study beginning at the time of diagnosis (2–3 years of age) will provide more detailed indications of disease characteristics and relationships with neurodevelopmental trajectories.

Therefore, in the present longitudinal study, we evaluated UF development over a 2-year interval in 2–3-year-old children with ASD or DD (controls). The following hypotheses were tested:

1. The UF is abnormal in children with ASD compared to children with DD.
2. Children with ASD exhibit changes in the early developmental growth trajectory of the UF.
3. Morphological growth of the UF is correlated with social interaction and social communication.

## Methods

### Participants

The data for this study were collected from the completed 2-year longitudinal neuroimaging project in our research team. The included data should comply with the following criteria: two neuroimaging data sets (baseline and follow-up), two consistent diagnoses, and high-quality scans. MRI data for both groups included two time points, including 37 children with ASD and 27 subjects in the DD group who matched their gender, age, developmental quotient (DQ) and intelligence quotient (IQ). Subjects with ASD were diagnosed depending on the diagnostic and statistical manual of mental disorders 4th edition, the text revision (DSM-IV-TR) criteria and standardized clinical assessments including the revised diagnostic interview for autism (ADI-R) by a licensed child and adolescent psychiatrist. The ASD group included children who meet the criteria of pervasive developmental disorders and excluded Rett’s Syndrome and childhood disintegrative disorder. Subjects in the DD group with mental retardation but did not meet ASD diagnostic criteria. Subjects with any type of systemic disease, history of head injury or genetic syndrome, neurological or psychiatric disease are ruled out.

### Clinical psychological assessment

(1) General survey: demographic data for all subjects, including birth history, past medical history, and family history, was examined based on a self-compiled scale.

(2) The ADI-R [43] is a widely used standardized and structured diagnostic tool for parent interviews and it is a semi-structured interview focusing on three domains: Communication (Verbal- VC and Non VerbalNVC), Reciprocal Social Interaction (RSI) and Repetitive Behaviour and Stereotyped Patterns (RBSP). Each item is scored from 0 (no impairment) to 2/3 (very severe delay/ deviance). In order to satisfy ADI-R criteria for autism, a child needs to score above cut-off in all three domains and to present with developmental concerns prior to the age of 3 years. ADI-R cut-off scores are 10 for RSI, 7 NVC, 8 for VC and 3 for RBSP.

(3) Both DQ and IQ were evaluated by a specially trained rater using the Bayley Scales of Infant Development-Chinese Version (BSID-C) [44] and the Peabody Picture Vocabulary Test (PPVT) [45]. BSID-C is a quantitative and standardized tool designed to assess the developmental level of infants, which could be utilized to generate the motor age and cognitive age. The DQ is a percentage of functional age (motor age + cognitive age) to double chronological age. PPVT is a receptive vocabulary test. Generally, the tested child refers to one of four pictures on a page named by an inspector. Total score can be converted to percentile level, mental age and IQ score.

### DTI scan parameters

A 3.0-T superconducting MRI system (Siemens, Germany) and planar echo sequence were employed for all scans. The scan parameters were as follows: diffusion sensitivity gradient directions = 30; diffusion sensitivity coefficient b value = 1000 s/mm2; scanning field = 230 mm × 230 mm; collection matrix = 128 × 128; slice thickness = 2.5 mm; echo time = 104 ms; repetition time = 9000 ms; scanning time = 5 min 8 s; slices for each scan = 60.

### DTI image analysis

**Fig. 1:**
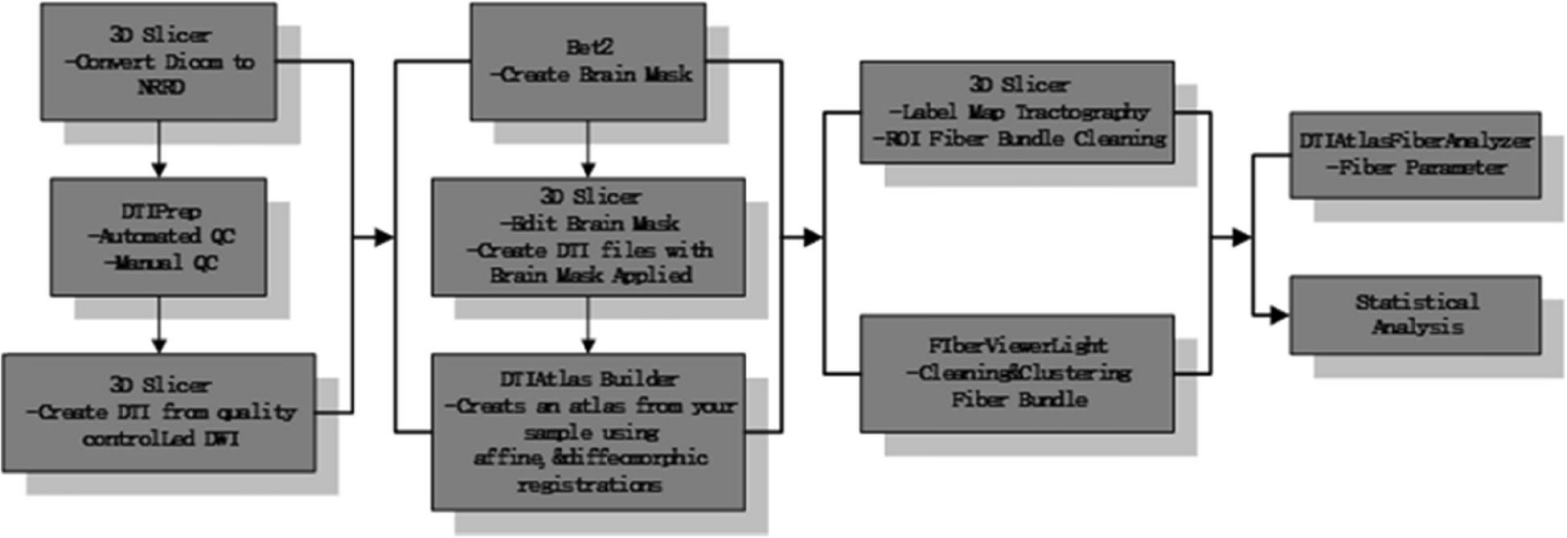
DTI data processing framework

**Fig. 2:**
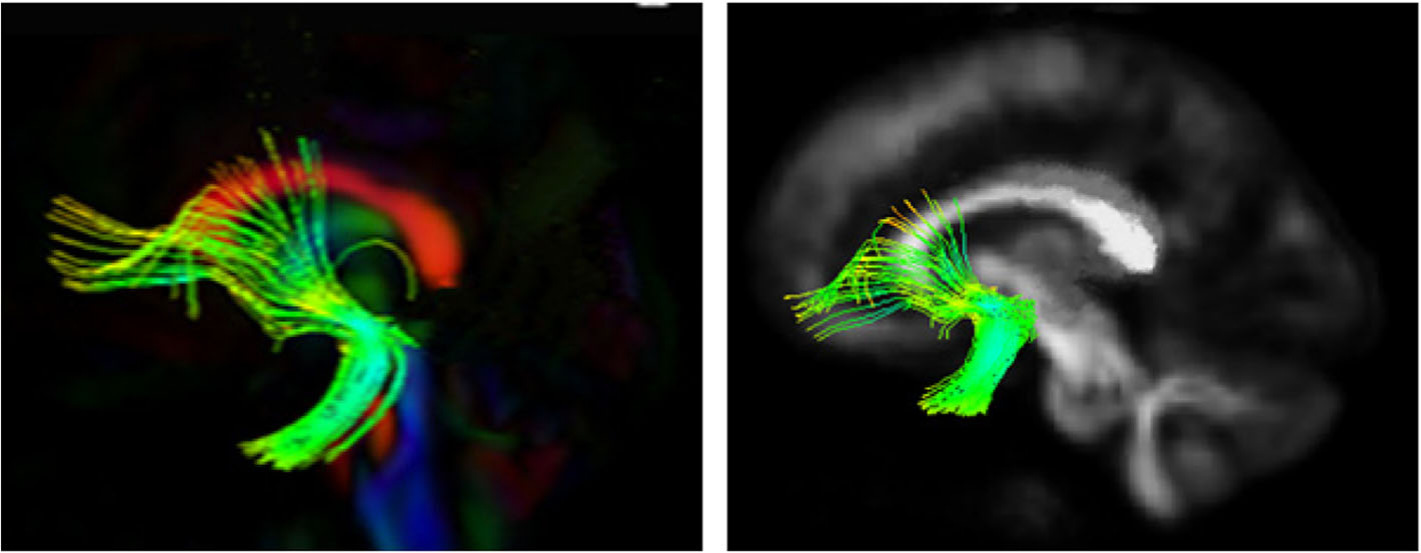
Right and left uncinate fasciculus tracing results

Our framework [46] for fiber bundle analysis of DTI images consists of four basic parts: (1) quality control using DTIPrep, (2) atlas creation using AutoSeg and DTIAtlasBuilder, (3) interactive fiber tractography using 3D slicer and FiberViewerLight, and (4) running DTIAtlasFiberAnalyzer to generate Diffusion property profiles and using SPSS to do statistical analysis. The tool’s work flow is illustrated in Fig. 1. All the tools referenced in our workflow description can be serve as stand-alone command line applications to facilitate scripting and grid computing, or to interact as external modules as part of the 3D Slicer. Although the first part of our framework is called quality control, it is critical that the process is assessed at each step of the framework to make sure the data analyzed is correct. The interactive fiber tractography was based on a template derived from the DTI scans for each group. The template was constructed using a deformation map and was used as an unbiased average atlas. Based on this compressed template, the bilateral UF areas were chosen as regions of interest (ROIs) to perform interactive fiber tractography for reconstruction of fiber tracts. Fiber tractography was achieved based on the ROI selection method for fiber tracts described by Catani [47]. The tool workflow is shown in Fig. 1, and the fiber reconstruction results are shown in Fig. 2.

### Statistical analysis

#### (1) Clinical demographics

Group differences in age, sex, DQ, IQ, and ADI-R scores at baseline and at 2-year follow-up were assessed using t-tests or chi-square tests.

#### (2) Cross-sectional analyses of UF at times 1 and 2

Two-tailed, independent, two-sample t-tests were used to test group differences in UF eigenvalues.

#### (3) UF development during the 2-year interval

For longitudinal analyses, the FA, MD, RD, and AD growth rates in the UF were calculated for each participant using the following equation: (time 2 UF eigenvalue-time 1 UF eigenvalue)/time 1 UF eigenvalue. Two-tailed, independent, two-sample t-tests were performed to test group differences in the growth rate of UF eigenvalues.

#### (4) Associations between UF development and social deficits

Associations between developmental changes in UF eigenvalues (time 2-time 1) and social deficits as measured by the ADI-R (time 2 score – time 1 score) in the ASD group were calculated using the Pearson’s correlation coefficient (r).

## Results

### Demographic data and clinical features

There were no significant group differences in sex ratio, age, DQ, or IQ. Compared to the DD group, the ASD group demonstrated significantly more severe social deficits, abnormalities in communication, and ritualistic-repetitive behaviors (RRBs) as determined by ADI-R scores, consistent with the differential diagnosis (Table 1).

**Table 1:** Participants: clinical demographics

	ASD group	DD group	*t/x*^*2*^	*P*
N	37	27		
Gender (male: female)	31:5	22:5	0.297	0.586
⊿ in age (months)	24.44 ± 4.48	24.74 ± 6.03	−0.224	0.824
Time 1				
Age (month)	30.86 ± 3.97	28.89 ± 5.23	1.701	0.094
DQ	69.19 ± 14.04	69.42 ± 17.42	−0.057	0.955
ADI-R				
Social Deficits	21.06 ± 6.16	12.22 ± 8.67	4.731	0.000***
Abnormalities in Communication	12.31 ± 3.71	7.30 ± 5.78	4.184	0.000***
RRB	4.53 ± 2.56	1.96 ± 1.89	4.385	0.000***
CARS	36.28 ± 4.12	27.33 ± 6.37	6.757	0.000***
Time 2				
Age (month)	55.31 ± 3.99	53.63 ± 4.09	1.632	0.108
IQ	85.56 ± 16.98	84.88 ± 25.61	0.114	0.910
ADI-R				
Social Deficits	17.83 ± 6.27	14.07 ± 8.53	2.017	0.048*
Abnormalities in Communication	12.56 ± 4.45	8.15 ± 5.36	3.563	0.001**
RRB	4.47 ± 2.01	2.74 ± 2.14	3.293	0.002**
CARS	34.11 ± 3.80	28.00 ± 8.51	3.835	0.000***

*ADI-R* Autism Diagnostic Interview Scale (Revised Edition), *BSID-CR* Bailey Infant Development Scale (China Urban Revision), *CARS* Child Autism Rating Scale; ⊿ in age: the interval between two scans * *P*<0.05; **P<0.01

### Cross-sectional analyses of UF at times 1 and 2

**Fig. 3:**
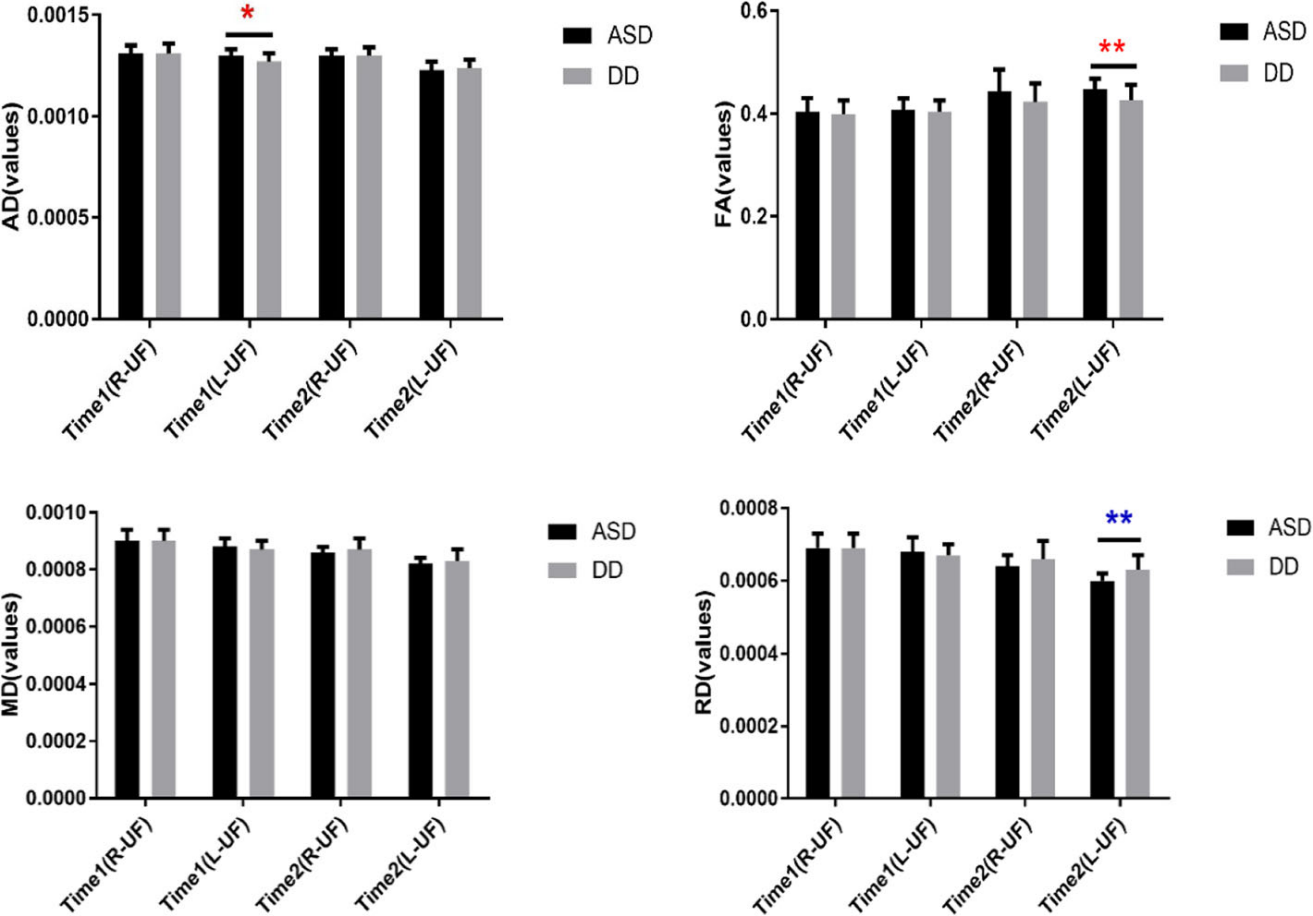
Cross-sectional analyses of uncinate fasciculus at Time 1 and Time 2. Note:ASD: Autism spectrum disorder; DD: Developmentally delayed; R-UF: Right-uncinate fasciculus; L-UF: Left-uncinate fasciculus; AD value: Axial diffusivity; FA value: fractional anisotropy; MD value: mean diffusivity; RD value: Radial diffusivity. *p < 0.05, **p < 0.01

Eigenvalues of the UF at times 1 and 2 are shown in Fig. 2. The AD values of the left UF were significantly larger in the ASD group than the DD group at time 1. At time 2, the FA value of the left UF was significantly higher (t = 3.307, *P* = 0.002) and RD value significantly lower in the ASD group compared to the DD group (t = − 3.07, *P* = 0.003) (Fig. 3).

### Group differences in UF development during the 2-year interval

**Fig. 4:**
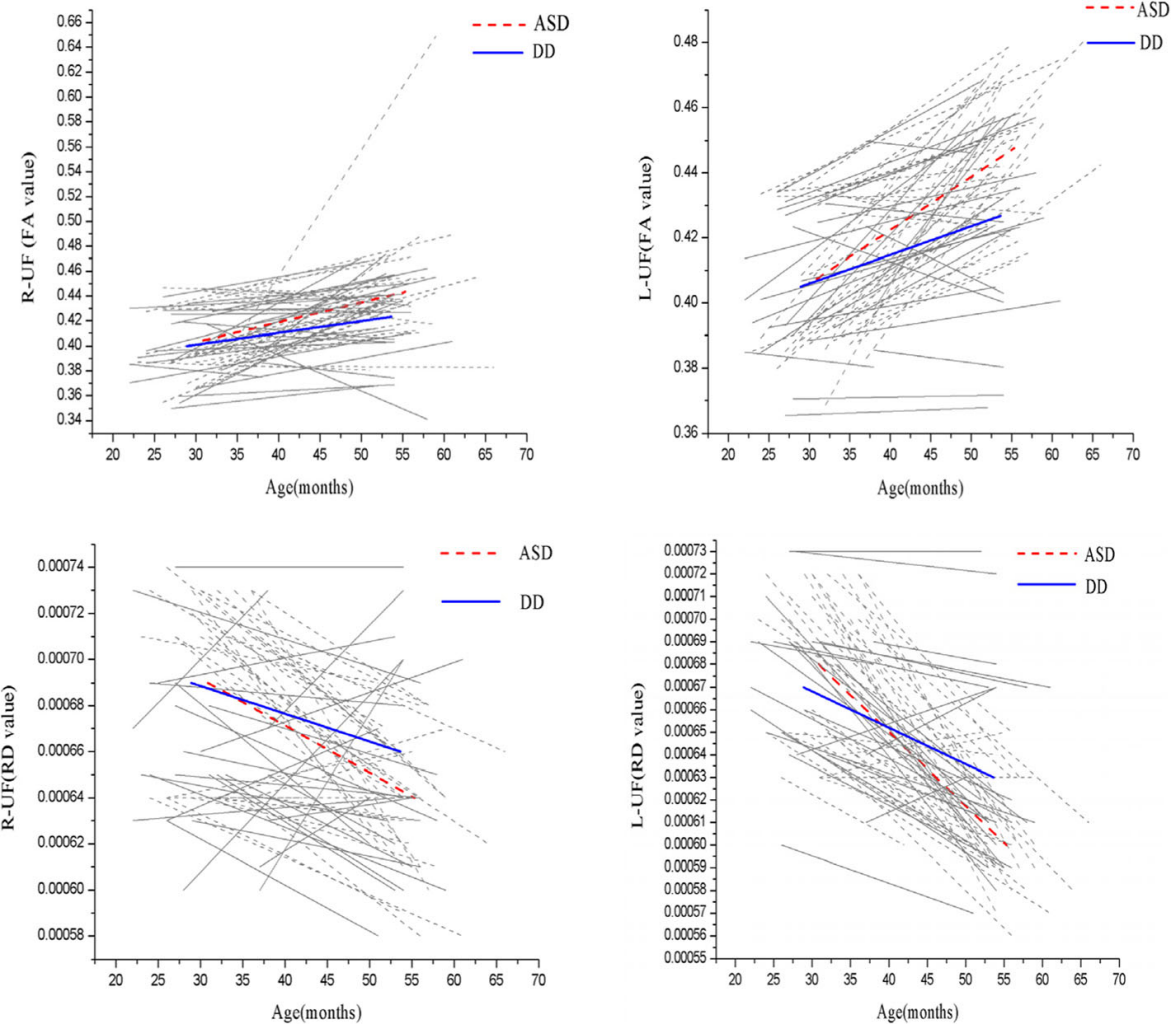
Trajectories of uncinate fasciculus growth (expressed by FA and RD values) in participants with autism spectrum disorder (ASD) (red and gray dotted lines) and developmentally delayed (DD) subjects (blue and gray solid lines). (For interpretation of the references to color in this figure legend, the reader is referred to the web version of this article)

The UF growth rate results are presented in Table 2. The left and right UF FA values increased by 10 and 10.2%, respectively, over the 2-year follow-up interval in the ASD group, compared to only 6.1 and 5.5%, respectively, in the DD group. The growth rate of the left UF FA value (t = 2.593, *P* = 0.012) was significantly greater in children with ASD compared to the DD subjects. Although the growth rate of the right UF FA value was higher in children with ASD than DD, the difference did not reach significance. Combining the growth rate over the interval between the two scans revealed a significant effect of diagnosis on the growth rate of the left UF FA value. The differences in AD, MD, and RD values were all significantly lower in the ASD group compared to the DD group (Table 2). Figure 4 presents individual and group-averaged growth trajectories of UF structures (expressed by FA and RD values) during the 2-year interval between scans for both groups.

**Table 2:** UF eigenvalues growth rate: ASD vs. DD

	Growth rate		*t-test*		*ANCOVA*^*a*^	
	ASD	DD	*t*	*p*	*F*	*P*
R-UF (AD)	−0.866 ± 3.115%	−0.377 ± 3.549%	−0.582	0.563	0.351	0.556
L-UF (AD)	−4.583 ± 4.344%	−2.773 ± 3.642%	−1.752	0.085	3.333	0.073
R-UF (FA)	10.039 ± 12.344%	6.093 ± 9.059%	1.401	0.166	1.995	0.163
L-UF (FA)	10.209 ± 7.801%	5.474 ± 6.232%	2.593	0.012*	7.183	0.009**
R-UF (MD)	−4.247 ± 3.827%	−2.814 ± 3.836%	−1.469	0.147	2.277	0.137
L-UF (MD)	−7.373 ± 4.360%	−4.824 ± 4.191%	−2.335	0.023*	6.201	0.016
R-UF (RD)	−7.119 ± 5.460%	−0.252 ± 7.254%	−4.290	0.000***	19.654	0.000***
L-UF (RD)	−10.731 ± 6.015%	−5.897 ± 5.582%	−3.254	0.002***	11.778	0.001**

*ASD* Autism spectrum disorder, *DD* Developmentally delayed, *R-UF* Right-uncinate fasciculus, *L-UF* Left-uncinate fasciculus, *AD value* Axial diffusivity, *FA value* Fractional anisotropy, *MD value* Mean diffusivity, *RD value* Radial diffusivity. ANCOVA^a^, The interval between two scans was included as covariate

*p < 0.05, ** *p* < 0.01, *** *p* < 0.001

### Correlations of social deficits and abnormalities in communication with UF development in the ASD group

**Fig. 5:**
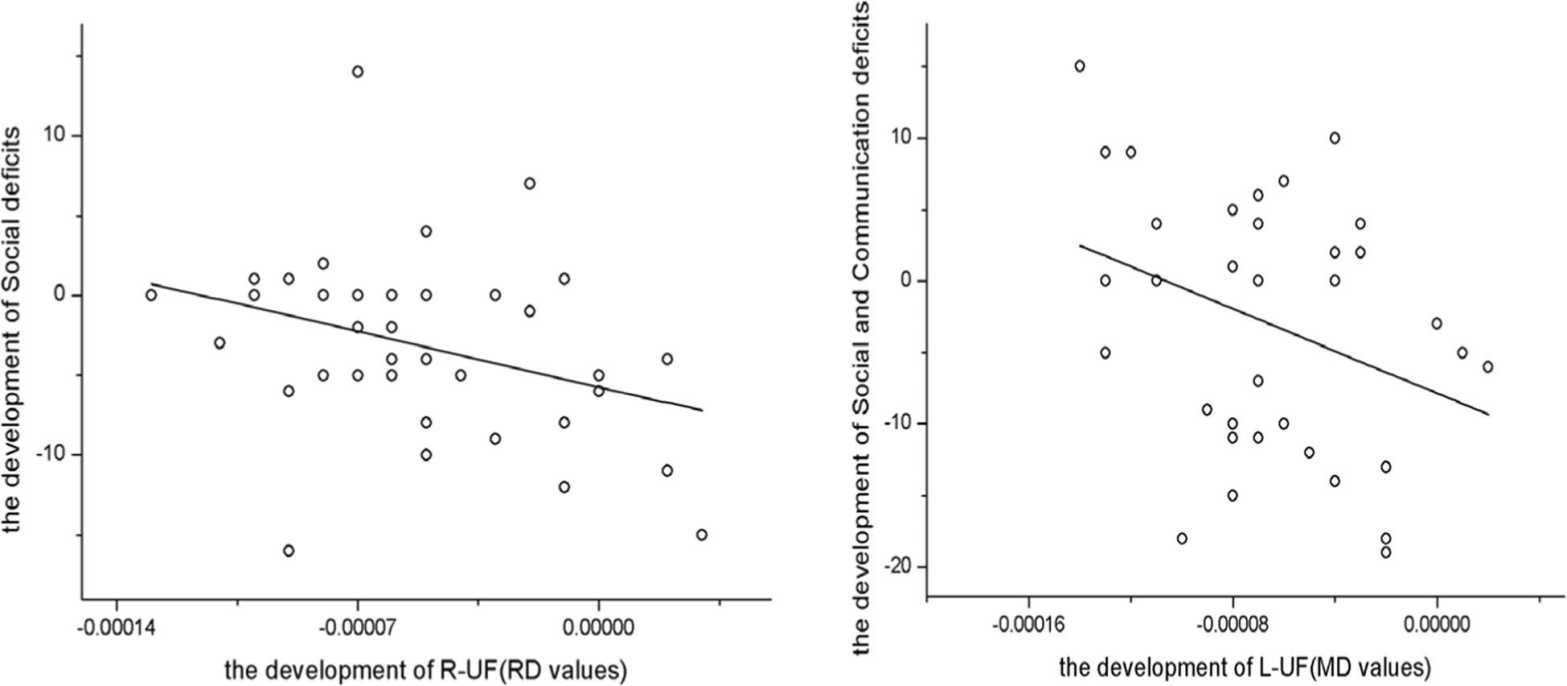
Correlation between the development of R-UF (RD values), L-UF (MD values) and Social Deficits

Pearson correlations were used to analyze association between the rise in social deficit measures over the 2-year interval and corresponding UF eigenvalues in children with ASD. Significant negative correlations were found between the rise in social deficit measures determined by the ADI-R and right UF RD values (r = − 0.336, *P* = 0.045) and between left UF MD values and the rises in ADI-R social deficits and abnormalities in communication (r = − 0.337, *P* = 0.045) (see Fig. 5). Alternatively, no significant relationships were found between the growth of social deficits scores and other UF eigenvalues (Table 3).

**Table 3:** Correlation between development of UF and Social Deficits and Abnormalities in Communication in ASD group

	ADI-R:Social Deficits		ADI-R:Social Deficits and Abnormalities in Communication	
	r	p	r	p
R-UF (AD)	0.060	0.730	−0.037	0.829
L-UF (AD)	0.030	0.863	−0.033	0.849
R-UF (FA)	−0.014	0.934	−0.004	0.982
L-UF (FA)	0.210	0.219	0.217	0.203
R-UF (MD)	−0.220	0.197	−0.247	0.146
L-UF (MD)	−0.308	0.068	−0.337	0.045*
R-UF (RD)	−0.336	0.045*	−0.308	0.068
L-UF (RD)	−0.297	0.078	−0.297	0.079

*ASD* Autism spectrum disorder, *DD* Developmentally delayed, *R-UF* Right-uncinate fasciculus, *L-UF* Left-uncinate fasciculus, *AD value* Axial diffusivity, *FA value* Fractional anisotropy, *MD value* Mean diffusivity, *RD value* Radial diffusivity. *p < 0.05, **p < 0.01

## Discussion

In many past DTI studies on the associations between neurodevelopmental trajectories and ASD deficits, FA was selected as the sole representative indicator of white matter microstructure. However, FA reflects only the ratio of the anisotropic component of water molecule diffusion to the entire diffusion tensor and does not provide an indicator of dispersion in all directions. Alternatively, an increase in MD may indicate poor white matter tissue quality while an increase in RD may indicate an abnormality in the microstructure of the fiber bundle or a lesion in the myelin sheath. Therefore, to further refine the white matter changes in all directions during development, the current study also incorporated MD, RD, and AD as indicators. We then examined the relationships between socio-affective deficits and structural OFC–amygdala connectivity in ASD by means of DTI. Measurements of FA, AD, MD, and RD in the bilateral UF revealed differences in structural connections between the prefrontal cortex and the amygdala and other structures, including the hippocampus. Furthermore, these structural abnormalities were correlated with the well described socio-affective deficits of ASD [3, 48–50].

An additional advantage of the current study design for examining white matter abnormalities in ASD is the acquisition of longitudinal measurements between about 2 and 5 years of age, corresponding to the initial developmental period after diagnosis. At baseline, the AD value of the left UF was significantly higher in the ASD group than the DD group, suggesting compensatory overgrowth of the left UF microstructure. At the two-year follow-up, the FA value of the left UF was significantly greater and the RD value of the left UF significantly lower in the ASD group, again suggesting compensatory overgrowth in ASD. Therefore, while children with both ASD and DD are likely to demonstrate excessive growth of the left UF, the magnitude of this abnormality is greater in ASD during early childhood.

Studies have shown increased FA values in younger individuals with ASD but decreased FA values in young adult and adult ASD patients. This age-dependence may account for inconsistent results across studies. Lee et al. [51] reported that the FA value of the temporal lobe was higher in ASD patients younger than 12 years old. Shukla et al. [52] compared the white matter skeletons of an ASD group and DD group and found that the FA values of ASD patients younger than 8 years old were greater while the MD values were lower. Cheng et al. [53] found that the FA values of ASD patients younger than 13 years old were elevated in some areas, such as the right parafollicular lobule, right frontal gyrus, and left superior longitudinal bundle. Mengotti et al. [54] studied 7 children with ASD of mean age less than 7 years and found that the MD values were decreased in the bilateral frontal cortex and the left corpus callosum. Therefore, despite variation among age groups, ASD patients younger than 7 or 8 exhibit greater FA values and lower MD values than age-matched controls, indicating excessive white matter growth. However, this pattern appears to be reversed after age 7–13 years. Longitudinal studies are needed to further confirm these conclusions. Overall, the ASD group in the current showed overgrowth in the left UF compared to the DD group, consistent with previous findings.

Studies of white matter development in childhood and adolescence as measured by DTI consistently demonstrate that FA increases with age throughout the white matter [55], presumably reflecting ongoing myelination during early childhood and increased organization and coherence of white matter tracts. Our results show that the UF bundles in both ASD and DD groups grew during the 2 years of follow-up, consistent with the findings of Olson and colleagues (2015) [8]. This rise in FA metrics of the UF suggests relative overgrowth in children with ASD compared to typically developing children or preschool DD children.

The UF connects the orbital aspect of the frontal lobe to the temporal pole and terminates in the amygdala, which is involved in processing information about the emotional significance of stimuli and in the generation of emotional expression [56]. Correlations were observed between UF development and both social deficits and communication abnormalities in the ASD group. Specifically, there was a significant negative correlation between the growth of the ADI-R social deficit score and the right UF RD value. That is, the smaller the RD value, the higher the social deficit score. Thus, our results strongly suggest a direct association between social deficits and the overgrowth of UF bundles in ASD. Our combined DTI and psychometric findings identify novel associations between socio-affective deficits and developmental trajectory of the UF in ASD. However, there are several methodological limitations to address. First, we studied a relatively small number of individuals with ASD or DD, which may have resulted in insufficient power to find moderation effects in several nodes of the UF. It is possible that a larger sample may reveal more robust effects and detectable associations in other nodes of this white matter structure. Therefore, the current results need to be replicated in a larger sample. Second, we focused on parents’ reports of socio-affective deficits in children with ASD. Future studies should include larger samples using behavioral measures of social deficits and emotional regulation. Third, we lacked a typical development (TD) control group to compare the results between ASD, DD and TD. In subsequent studies we will enroll in the TD group. Finally, it is known that the UF is one of the latest developing tracts, with a maturational peak in the third decade of life [8]. This makes it a potentially interesting structure to assess the efficacy of treatments such as socio-affective skill development over a longer time span through longitudinal studies.

## Conclusions

Our findings reveal UF overgrowth during development in ASD. Moreover, this overgrowth was correlated with social and communication deficit severity, suggesting a direct contribution to pathogenesis and (or) symptom expression in young ASD patients.

## Data Availability

The DTI scans used and datasets analyzed for the current study are available from the corresponding author on reasonable request.

## Abbreviations

AD: Axial diffusivity
ADI-R: Autism Diagnostic Interview-Revised
ASD: Autism Spectrum Disorder
DD: Developmentally Delayed
DQ: developmental quotient
DSM-IV-TR: Diagnostic and Statistical Manual of Mental Disorders, 4th ed., text revision
DTI: Diffusion Tensor Imaging
FA: Fractional Anisotropy
IQ: Intelligence quotient
MD: Mean Diffusivity
MRI: Magnetic resonance imaging
NVC: None Verbal Communication
OFC: Orbitofrontal Cortex
RBSP: Repetitive Behaviour and Stereotyped Patterns
RD: Radial diffusivity
Rs-fMRI: Resting state functional magnetic resonance imaging
RSI: Reciprocal Social Interaction
UF: Uncinate Fasciculus
VC: Verbal Communication
